# Optimizing Paclitaxel Oral Absorption and Bioavailability: TPGS Co-Coating via Supercritical Anti-Solvent Fluidized Bed Technology

**DOI:** 10.3390/ph17040412

**Published:** 2024-03-25

**Authors:** Zicheng Zhong, Yanling Lan, Jinxing Chen, Lu Ping, Xuchun Li, Qing Wang, Xiaodong Zhuang, Zhenwen Qiu, Tianhui Yuan, Qiupin Guo, Long Xi, Qingguo Li, Dandong Luo

**Affiliations:** 1 School of Pharmaceutical Sciences, Guangzhou University of Chinese Medicine, 232 University City Ring Road East, Panyu District, Guangzhou 510006, China; zhongzicheng168@163.com (Z.Z.); yanling.lan0@outlook.com (Y.L.); 13535317795@163.com (J.C.); 13435751755@163.com (X.L.); 13208599472@163.com (Q.W.); xilong@gzucm.edu.cn (L.X.); 2 The Third Affiliated Hospital of Guangzhou University of Chinese Medicine, Guangzhou 510378, China; 3 The First Affiliated Hospital of Guangzhou University of Chinese Medicine, Guangzhou 510405, China; pinglu@stu.gzucm.edu.cn (L.P.); qzhenwen@gzucm.edu.cn (Z.Q.); laura.yth@hotmail.com (T.Y.); 4 Division of Infection and Immunity, University College London, London OX3 7FZ, UK; xiaodong.zhuang@ucl.ac.uk; 5 Drug Non-Clinical Evaluation and Research Center of Guangzhou General Pharmaceutical Research Institute, Guangzhou 510240, China; 13427595023@163.com

**Keywords:** paclitaxel, supercritical anti-solvent fluidized bed, TPGS, oral absorption, bioavailability

## Abstract

Supercritical anti-solvent fluidized bed (SAS-FB) coating technology has the advantages of reducing particle size, preventing high surface energy particle aggregation, improving the dissolution performance and bioavailability of insoluble drugs. The poor solubility of Biopharmaceutics Classification System (BCS) class IV drugs poses challenges in achieving optimal bioavailability. Numerous anti-cancer drugs including paclitaxel (PTX) belong to the BCS class IV, hindering their therapeutic efficacy. To address this concern, our study explored SAS-FB technology to coat PTX with D-α-tocopherol polyethylene glycol 1000 succinate (TPGS) onto lactose. Under our optimized conditions, we achieved a PTX coating efficiency of 96.8%. Further characterization confirmed the crystalline state of PTX in the lactose surface coating by scanning electron microscopy and X-ray powder diffraction. Dissolution studies indicated that SAS-FB processed samples release over 95% of the drug within 1 min. Moreover, cell transmembrane transport assays demonstrated that SAS-FB processed PTX samples co-coated with TPGS had an enhanced PTX internalization into cells and a higher permeability coefficient compared to those without TPGS. Finally, compared to unprocessed PTX, SAS-FB (TPGS) and SAS-FB processed samples showed a 2.66- and 1.49-fold increase in oral bioavailability in vivo, respectively. Our study highlights the efficacy of SAS-FB co-coating for PTX and TPGS as a promising strategy to overcome bioavailability challenges inherent in BCS class IV drugs. Our approach holds broader implications for enhancing the performance of similarly classified medications.

## 1. Introduction

Many anti-cancer medications fall within the category of Biopharmaceutics Classification System (BCS) class IV drugs, which are characterized by both low solubility and low permeability, hindering their therapeutic efficacy. The limited solubility of these drugs can affect their dissolution in the gastrointestinal tract, potentially leading to challenges in absorption and, consequently, reduced bioavailability. Paclitaxel (PTX) is a natural diterpene and a typical BCS class IV drug [1] which has been widely used in treating diverse malignancies including breast, bladder, melanoma, esophageal, ovarian, and lung cancers [2,3,4,5,6,7]. However, PTX lacks ionizable functional groups, leading to major obstacles in therapeutic applications due to its extremely low aqueous solubility (0.3 μg/mL) and poor permeability (5.2 × 10^−6^ cm/s) [8,9]. Due to its susceptibility as a substrate for the drug efflux pump P-glycoprotein (P-gp) with high affinity [10], PTX requires intravenous administration in clinical settings [11]. However, this mode of administration comes with typical disadvantages including severe allergic reactions, myelosuppression, compromised patient compliance, neurotoxicity, and heightened infection risk [12]. Given these challenges, exploring alternative administration routes becomes imperative to mitigate associated adverse effects and enhance therapeutic outcomes.

Oral administration offers patients the convenience of self-medication while alleviating the discomfort associated with injections. This method not only enhances patient compliance but also reduces formulation costs. Therefore, choosing the oral route for administering PTX holds promise. However, the current oral bioavailability of PTX is below 10%, limiting its therapeutic efficacy [13]. For BCS class IV drugs like PTX, solubility and permeability significantly influence absorption. Improving oral PTX formulations to enhance bioavailability involves strategies aiming at increasing solubility and permeability which are crucial in determining absorption efficiency. Post oral administration, drugs are dissolved in the gastrointestinal tract before entering systemic circulation through intestinal epithelial cells [14], However, drug absorption can be impacted by limited residence time and specific absorption sites within the gastrointestinal tract, coupled with variations in cellular structure and function across its different parts. Consequently, considerable efforts have been made to enhance solubility, permeability, and overall bioavailability of oral PTX administration. Numerous drug delivery systems have emerged, including polymeric micelles [8,15]. Chen et al. [16] developed a novel chitosan derivative (GA-CS-TPGS copolymer) and constructed PTX micelles. The PTX micelles enhanced bioadhesion, solubility, and permeability and inhibited P-gp efflux and drug metabolism in liver. The bioavailability of PTX was improved approximately 3.80-fold by the PTX Micelles. Liu et al. [17] designed a novel chitosan-thioglycolic acid-Pluronic F127 (CSTGA-PF) liposome system for PTX oral delivery. The optimized formulation of PTX-loaded CSTGA-PF liposomes showed zeta potential of 50.2 mV and particle size of 121.4 nm. PTX could be slowly released in gastrointestinal fluid, prolonging its residence time on the surface of the intestinal mucosa and increasing its absorption in the intestine. Godara et al. [18] designed a PTX-loaded lipid-PLGA hybrid nanoparticles (NPs) for oral delivery. The development of NPs was aimed to utilize the advantage of structural integrity of lipid in the shell and hybrid NPs containing PLGA core. Human serum albumin (HSA), TPGS, pluronic 68 (F68), and polyvinyl alcohol (PVA) were used as stabilizers. The particle size of NPs was 150–400 nm. Pharmacokinetic studies have shown that HAS-based nanoparticles have a higher Cmax and AUC0-infinity. Through the co-modification of thiolated chitosan materials with polylysine (PL) and polylactide (PLA), Du et al. [19] developed a chitosan-based multifunctional nanoparticle (PY-CS-PLA) for the oral administration of paclitaxel. In vitro studies showed that the encapsulated paclitaxel nanoparticles were effective in enhancing the uptake of paclitaxel by Caco-2 cells. Pharmacokinetic results showed that the oral bioavailability of paclitaxel nanoparticles in rats was increased by 5.63-fold. Ding et al. [20] developed a new oral delivery method for paclitaxel by combining a phospholipid-drug complex (PLDC) with a Selfnanoemulsifying drug delivery system (SNEDDS). PLDC-SNEDDS forms nanoemulsions with an average particle size of 30 nm in water. In vivo oral absorption studies showed that the bioavailability of PTX-PLDC-SNEDDS was 2.13 and 3.42 times higher than that of paclitaxel and PTX-PLDC solution, respectively. Nevertheless, these methods often involve complex processes and formulations, utilizing substantial amounts of medicinal excipients, posing drawbacks in practical implementation.

In recent decades, supercritical fluid technology (SCF) has emerged as a promising method for producing sub-micro- and nano-functional materials. OF the SCF processes, supercritical anti-solvent (SAS) stands out for its ability to generate drug nanoparticles/nanocrystals and co-precipitate drugs and excipients [21,22,23,24]. SAS capitalizes on the high diffusivity of SCF compared to liquids, prompting the rapid supersaturation and precipitation of solutes into microparticles and nanoparticles when SCF diffuses into an organic solvent [25]. However, these particles tend to agglomerate due to their high surface energy, leading to poor flowability and difficulties in subsequent processing, limiting the application of SAS technology. To address this issue, SAS-precipitated drugs were combined with a coating process inside a dynamic fluidized bed (FB) to develop highly dispersed drug-loaded particles [26,27,28]. This approach, termed the supercritical anti-solvent fluidized bed (SAS-FB) procedure, achieves one-step simultaneous precipitation and coating, significantly enhancing drug dissolution and bioavailability.

The majority of research on SAS-FB has focused on improving the solubility of BCS class II drugs, which are characterized by high permeability but low solubility. Through SAS-FB, uniform curcumin precipitation onto lactose surfaces with coating efficiencies ranging from 71.0% to 93.3% and particle sizes between 0.41 and 12.08 μm can be achieved [29]. SAS-FB-processed Sirolimus demonstrated enhanced solubility and oral bioavailability [26], with additional improvements achieved by incorporating excipients into the SAS-FB process for co-precipitation with the drug [30]. In addition, flavonoids such as luteolin, naringenin, and dihydromyricetin achieved particle size reduction, solubility enhancement, and antioxidant activity increase through the SAS-FB process [27]. Naringin, coated onto micronized matrix particles using SAS-FB for dry powder inhaler delivery of nanomedicines, exhibited an amorphous structure at an average size of approximately 130 nm, demonstrating superior in vivo plasmatic and pulmonary bioavailability [28]. However, despite SAS-FB showing promise in enhancing drug properties, its application for improving the solubility and permeability of low-permeability drugs remains unexplored.

D-α-tocopherol polyethyleneglycol 1000 succinate (TPGS) is a safe, non-toxic, and biocompatible pharmaceutical excipient, drawing significant attention in the field for its ability to enhance drug stability, promote absorption, and exhibit excellent surface activity. When combined with drugs, TPGS enhances solubility and significantly improves permeability in drugs that are typically difficult to dissolve. This synergistic effect not only extends the duration of drug action but also markedly enhances oral bioavailability. Recent research has shown TPGS is capable of counteracting P-glycoprotein (P-gp)-mediated multidrug resistance while reducing the body’s drug efflux. Studies reveal that TPGS functions below the critical micelle concentration, competitively hindering substrate binding, altering membrane fluidity, and inhibiting the ATPase function of efflux pumps. These mechanisms collectively impede drug efflux from P-gp, substantially augmenting the therapeutic effectiveness of anti-cancer medications [31,32,33,34,35,36]. In addition, functional excipients Gelucire 44/14 and Pluronic F68 also have an inhibitory effect on P-gp [37,38]. For the purpose of screening excipients and optimizing formulations, TPGS, Gelucire 44/14, and Pluronic F68 were selected for process optimization and dissolution comparison.

Our study focused on co-precipitating PTX with TPGS onto lactose using the SAS-FB procedure, using commercial medicinal lactose (Tablettose^®^70) as an oral delivery matrix and for fluidization purposes. We conducted a systematic examination on the SAS-FB process parameters including temperature, pressure, solution concentration, solvent, and excipient, with the aim of optimizing the process to develop a novel PTX oral formulation to achieve high drug loading, improved solubility, and enhanced permeability. Physicochemical characterizations were performed on the SAS-FB processed samples to analyze drug morphology, particle size, crystalline state, and interactions within the PTX-TPGS-lactose system. To assess the efficacy of the developed formulation, in vitro dissolution studies, cellular uptake analyses, cellular transmembrane transport assays, and in vivo pharmacokinetic evaluations were conducted. Our results demonstrate that the co-precipitation of PTX and TPGS via the SAS-FB process effectively enhances PTX dissolution, permeability, and oral bioavailability.

## 2. Results and Discussion

### 2.1. Drug Loading of SAS-FB Products

**Figure 1:**
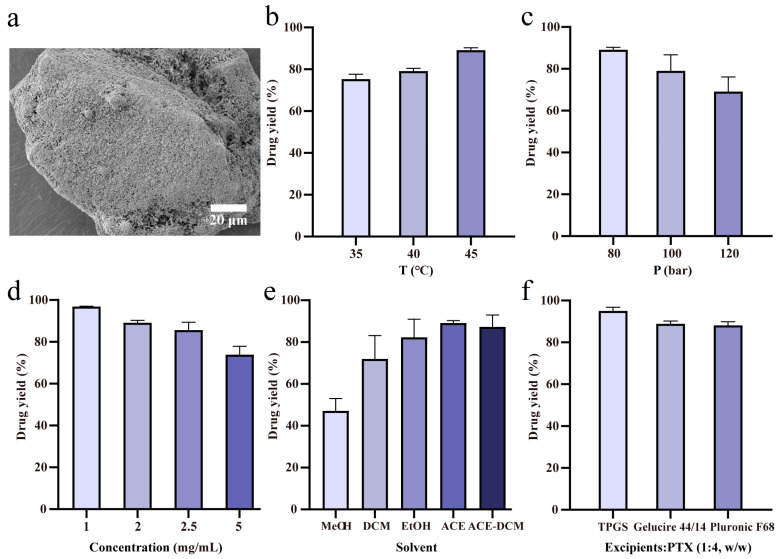
Effect of various conditions and excipients on the yield of Paclitaxel (PTX). (**a**) Schematic diagram illustrating the coating of PTX and excipients onto lactose particles; (**b**) temperature (tests 1–3); (**c**) pressure (tests 3–5); (**d**) drug concentration (tests 12, 3, 6 and 7); (**e**) solvent (tests 8–10, 3 and 11); and (**f**) excipients (tests 13–15) were evaluated for their effects on drug yield (*n* = 3, mean ± SD). Conditions for individual tests are shown in Table 1.

Coating efficiency holds significant importance in pharmaceutical manufacturing. We evaluated coating efficiency of PTX using the SAS-FB process under various parameters which include three temperature levels (35, 40, 45 °C), three pressure levels (80, 100, 120 bar), four drug concentrations (1, 2, 2.5, 5 mg/mL), five solvents (MeOH, DCM, EtOH, Acetone, ACE-DCM), and three excipients (TPGS, Pluronic F68, Gelucire 44/14). The analysis revealed that these operating conditions can impact the coating efficiency at different levels. The schematic diagram of the coating of PTX and excipients onto lactose particles is shown in Figure 1a.

The SAS-FB process was evaluated under temperature variations at 35, 40, and 45 °C (Figure 1b), as well as pressure changes at 80, 100, and 120 bar (Figure 1c), while all other process conditions remained constant (C_drug_ = 2 mg/mL, f_drug flow rate_ = 1.5 mL/min, f_CO2_ = 32 g/min, M_lactose carrier_ = 2 g). As the temperature increased from 35 to 45 °C, there was a corresponding increase in coating efficiency from 75.3% to 89.1%, depicting a consistent upward trend. Conversely, with pressure increasing from 80 to 120 bar, the coating efficiency decreased from 89.1% to 69.1%, indicating a clear declining pattern. The temperature and pressure’s influence derived from its impact on the supercritical CO_2_ (SC-CO_2_) density. It was reported that the solubility of PTX in SC-CO_2_ with a density of 0.0140–0.0214 mol/cm^3^ is only 1.10–6.32 × 10^6^ [39]. However, the density of SC-CO_2_ at 45 °C and 80 bar is 0.0056 mol/cm^3^, far below the reported range. Therefore, PTX had lower solubility and higher yield in SC-CO_2_ at 45 °C and 80 bar. At lower pressures (*p* = 80 bar) and higher temperatures (T = 45 °C), the density of SC-CO_2_ decreased, reducing its ability to dissolve the drug. This condition facilitated enhanced mass transfer between the solvent and SC-CO_2_, increasing the solution’s supersaturation degree and leading to increased solid drug precipitation from the sc-fluid. In contrast, higher pressures (*p* = 120 bar) caused an increase in SC-CO_2_ density, enhancing its drug-dissolving capacity and reducing the solution’s saturation rate. This resulted in decreased solid drug precipitation from the sc-fluid, therefore contributing to a decrease in coating efficiency. Screening experiments confirmed that operating at 45 °C and 80 bar represented the optimal conditions for the SAS-FB process within this study system. Consistent trends were observed for PTX coating efficiency compared to previous findings with sirolimus and three flavonoids [26,27]. These findings established that the optimal conditions for the SAS-FB process corresponded to the low-density region of SC-CO_2_ at high temperature and low pressure, facilitating drug precipitation.

In the field of supercritical fluids, the concentration of the drug solution can significantly impact the yield of the SAS process. Our study specifically evaluated varying concentrations of PTX dissolved in acetone solution at 1, 2, 2.5, and 5 mg/mL, while maintaining fixed SAS-FB operational conditions (T = 45 °C, *p* = 80 bar, f_drug flow rate_ = 1.5 mL/min, f_CO2_ = 32 g/min, M_lactose carrier_ = 2 g). The observed coating efficiencies were 96.8%, 89.1%, 85.6%, and 73.8%, respectively (Figure 1d). These results demonstrated an inverse relationship between PTX coating efficiency and its concentration in the solution. Specifically, the lowest drug concentration at 1.0 mg/mL exhibited the highest coating efficiency at 96.8%. However, as the concentration increased, there was a gradual decrease in coating efficiency.

In the SAS process, the interactions among different solvents, solutes, and supercritical CO_2_ play an important role in determining the yield of precipitated drugs [21]. Our study evaluated five distinct solvents: methanol (MeOH), dichloromethane (DCM), ethanol (EtOH), acetone (ACE), and a 50:50 mixture of acetone–dichloromethane (ACE-DCM), all at operating conditions of 45 °C, 80 bar pressure, 1.5 mL/min PTX solution, 32 g/min CO_2_ flow rates, and 2 g lactose carrier. The PTX solution concentration was kept constant at 2 mg/mL. The resulting coating efficiencies for these solvents were 47.2%, 71.9%, 82.3%, 89.1%, and 87.3%, respectively (Figure 1e). Notably, when acetone was used as the solvent for preparing the drug solution, it yielded the highest coating efficiency at 89.1%. Conversely, methanol resulted in the lowest coating efficiency of 47.2%. This variation in coating efficiency was influenced by the choice of solvent, primarily linked to the physicochemical properties of PTX and the diverse solvents, as well as the solubility of PTX in both the solvents and supercritical CO_2_. Different solvents possess unique physical and chemical characteristics, such as surface tension, viscosity, density, and dielectric constant. Hence, the selection of the solvent plays a critical role when conducting investigations employing the SAS-FB process [27].

The oral absorption challenges faced by the BCS class IV drug PTX cannot be solely addressed by enhancing its dissolution while improving drug permeability is equally crucial. To enhance permeability, a strategy involving the co-precipitation of PTX and TPGS anti-solvent on lactose surface was explored under optimized conditions. TPGS was selected because of its capacity to enhance drug stability, facilitate permeation and absorption, reverse P-gp-mediated multidrug resistance, and reduce drug efflux from the body [31,32,33,35]. Further, our study investigated coating efficiency when co-coated with other excipients, including Gelucire 44/14 and Pluronic F68, both known to inhibit P-gp [37,38]. The findings demonstrated coating efficiencies of 95.0% for SAS-FB (TPGS), 88.9% for SAS-FB (Gelucire 44/14), and 88.1% for SAS-FB (Pluronic F68) (Figure 1f). Notably, SAS-FB (TPGS) exhibited the highest drug yield of these methods. This outcome suggests a broad applicability of SAS-FB technology in co-precipitating drugs with excipients in pharmaceutical formulation.

### 2.2. In Vitro Dissolution

**Figure 2:**
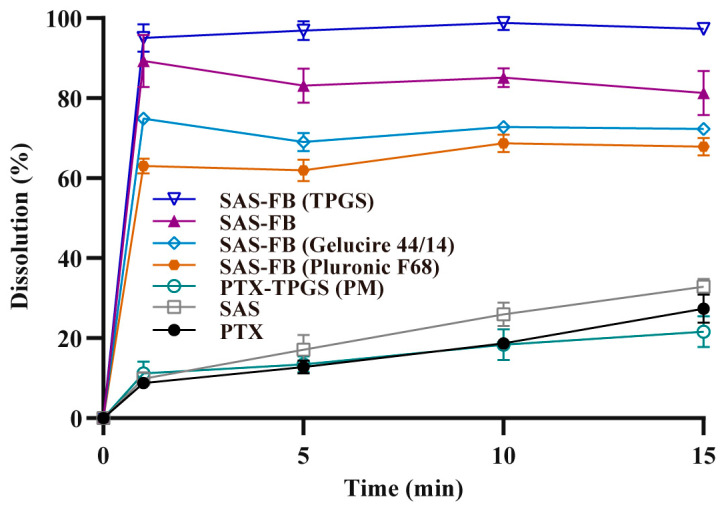
Dissolution profiles of raw PTX, SAS (test 18), PTX-TPGS (PM), SAS-FB (Pluronic F68) (test 15), SAS-FB (Gelucire 44/14) (test 14), SAS-FB (test 12), SAS-FB (TPGS) (test 13) in pH 6.8 dissolution media (*n* = 3, mean ± SD). Conditions for individual tests are shown in Table 1.

The dissolution experiments using various samples including PTX, SAS, PTX-TPGS (physical mixture, PM), SAS-FB, SAS-FB (TPGS), SAS-FB (Gelucire 44/14), and SAS-FB (Pluronic F68), were carried out in a pH 6.8 dissolution medium (Figure 2). Results indicated that within 1 min, the dissolution percentages for SAS-FB, SAS-FB (TPGS), SAS-FB (Gelucire 44/14), and SAS-FB (Pluronic F68) were 89.0%, 95.0%, 74.8%, and 63.0%, respectively. Importantly, SAS-FB (TPGS) exhibited the highest dissolution at 95.0%, approximately 10.8 times higher than PTX and 9.6 times higher than SAS. Over 15 min, SAS-FB (TPGS) showed a dissolution of 97.3%. This indicated that SAS-FB (TPGS) significantly outperformed other samples in terms of in vitro dissolution at both 1 and 15 min. It even surpassed previously reported PTX solid dispersion and micelle formulations [8,16] with a simpler formulation and reduced excipient dosage, which highlights the advantage of the SAS-FB process. The SAS-FB technique combines the preparation characteristics of SAS and FB, where precipitated drug particles are adsorbed and distributed on the surface of fluidized carrier lactose in a dispersed/unaggregated state, avoiding the further aggregation and growth of precipitated drug particles into large particles. According to the Ostwald–Freundlich and Noyes–Whitney equations, reducing the particle size of insoluble drugs can effectively improve their solubility and dissolution rates. In addition, by comparing the in vitro dissolution of PTX-TPGS (PM) with that of SAS-FB (TPGS) samples, we found that the co-precipitation of TPGS and PTX co-coated on the surface of a lactose carrier could better utilize the effects of TPGS in enhancing drug stability and increasing drug solubility. However, there was no difference in the dissolution of PTX-TPGS (PM) samples compared to raw PTX.

**Figure 3:**
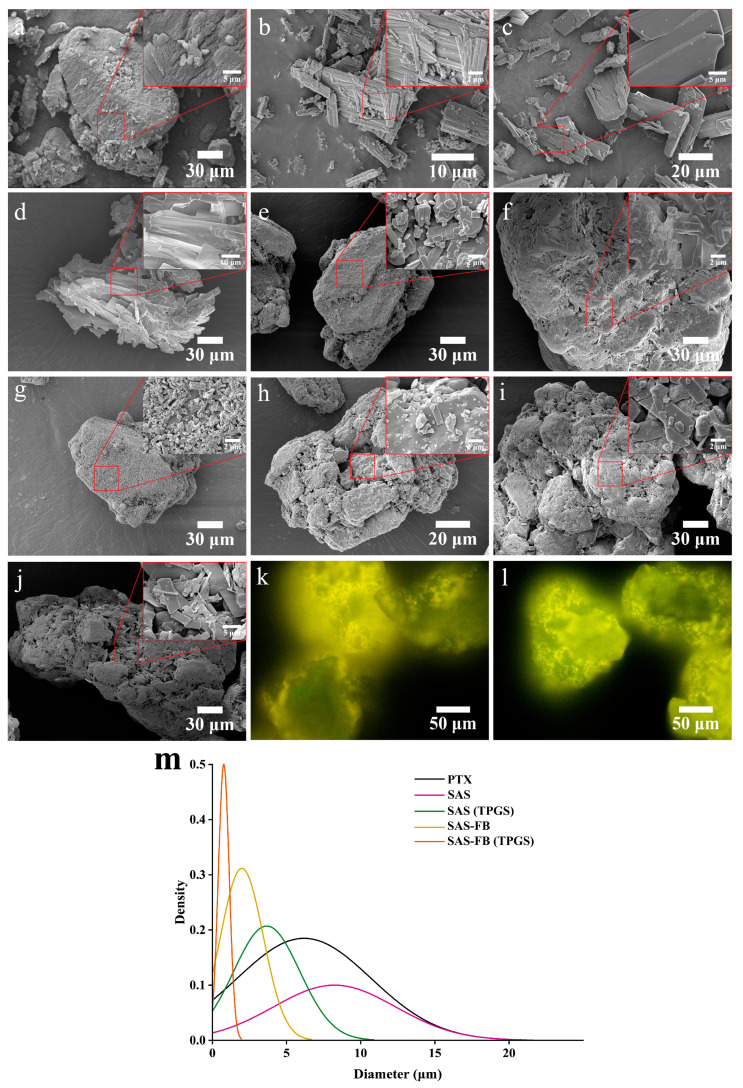
Scanning electron microscopic images of (**a**) lactose × 1000 (test 21); (**b**) untreated paclitaxel (PTX) × 5000; (**c**) SAS × 2000 (test 18); (**d**) SAS (TPGS) × 1000 (test 19); (**e**) SAS-FB × 1000 (test 12); (**f**) SAS-FB (TPGS) × 1000 (test 13); (**g**) SAS-FB (MeOH) × 1000 (test 8); (**h**) SAS-FB (EtOH) × 2000 (test 10); (**i**) SAS-FB (DCM) × 1000 (test 9); and (**j**) SAS-FB (ACE-DCM) × 1000 (test 11). Fluorescence microscopy of (**k**) SAS-FB (Cou-6) (test 16) and (**l**) SAS-FB (Cou-6, TPGS) (test 17). (**m**) Particle size distribution derived from the analysis of scanning electron microscope (SEM) images. Conditions for individual tests are shown in Table 1.

Using SAS-FB process to co-precipitate PTX and TPGS onto the carrier enhanced PTX dissolution. Conversely, PTX co-precipitated with Gelucire 44/14 or Pluronic F68 showed reduced dissolution rates. The SAS method, due to crystalloid particles and larger particle size (Figure 3c,m), failed to enhance dissolution. SEM images revealed that PTX particles in the SAS-FB sample were smaller and evenly dispersed on lactose surface, preventing crystal growth and aggregation. This result suggests that the SAS-FB process holds promise in resolving challenges posed by insoluble BCS class II and IV drugs, offering valuable support for handling such compounds [26,27,28,30]. Additionally, considering that excipients can exert their inhibitory effect on P-gp below the critical micelle concentration (CMC) [32], the SAS-FB and SAS-FB (TPGS) samples, which demonstrated high drug loading capacity and superior in vitro dissolution, were selected for further studies.

### 2.3. Particle Characterization

The physicochemical properties of PTX, SAS (test 18), SAS (TPGS) (test 19), SAS (Cou-6) (test 20), SAS-FB (test 12), SAS-FB (TPGS) (test 13), SAS-FB (Cou-6) (test 16), SAS-FB (Cou-6, TPGS) (test 17), and lactose (test 21) were characterized using SEM, FM, XRPD, DSC, FT-IR, and DRS.

The morphology of the samples was evaluated using SEM, as shown in Figure 3a–j. The particle size distribution is illustrated in Figure 3m and Figure S1. Lactose and unprocessed PTX were served as controls (Figure 3a,b). The results revealed a crystalline state for all samples. SAS and SAS (TPGS) appeared as bulk and flake formations (Figure 3c,d). In contrast, the SAS-FB sample displayed short clumps uniformly dispersed on the lactose surface (Figure 3e), with an average particle size 1.9 μm smaller than SAS particles (8.2 μm). Upon the addition of TPGS, which co-precipitated with PTX, PTX and TPGS formed a mutual mosaic structure, coating the lactose surface uniformly (Figure 3f). Importantly, TPGS not only affected crystal morphology but also further reduced particle size (Figure 3m).

Interestingly, the choice of solvent not only influences the coating efficiency but also significantly impacts the crystalline morphology and particle size of PTX when coated onto the lactose surface. Acetone, methanol, ethanol, dichloromethane, and ACE-DCM were used as solvents to produce SAS-FB samples. The morphologies observed for PTX on the lactose surface included small blocks, short columns, fragments, flakes, and a mixed state of block and rod shapes, respectively (Figure 3e,g–j). Their average particle sizes were 1.98, 0.69, 0.63, 3.76, and 2.75 μm, respectively (Figure S1). The particle sizes of samples prepared with these five solvents arranged in descending order were: dichloromethane > acetone–dichloromethane (50/50) > acetone > methanol > ethanol.

The co-precipitation of PTX and Cou-6 on the lactose surface exhibits a distinct fluorescence property. This fluorescence property proves beneficial for the visualization and analysis of PTX co-precipitated on the lactose surface using FM (Figure 3k,l). The co-precipitation of PTX and Cou-6 results in a yellow-green fluorescence covering the surface of lactose. This fluorescence indicates successful Cou-6 precipitation onto specific binding sites on the lactose surface.

**Figure 4:**
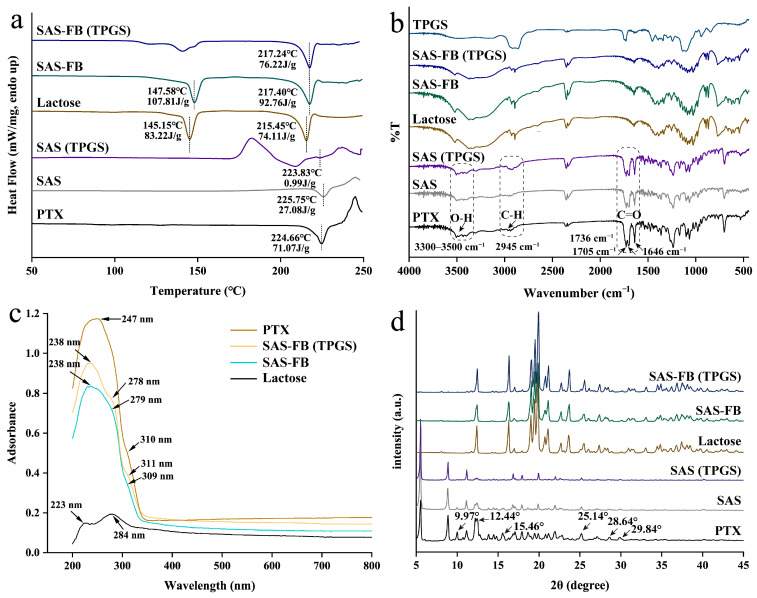
Physicochemical characterizations of processed PTX. (**a**) Differential scanning calorimeter (DSC) heat flow curves of raw PTX, SAS (test 18); SAS (TPGS) (test 19); lactose, SAS-FB (test 12); and SAS-FB (TPGS) (test 13). (**b**) Fourier transform infrared spectroscopy (FT-IR) spectra of raw PTX, SAS (test 18); SAS (TPGS) (test 19); lactose, SAS-FB (test 12); SAS-FB (TPGS) (test 13); and TPGS. (**c**) Diffuse reflectance spectroscopy (DRS) spectrum of raw PTX, SAS-FB (TPGS) (test 13); SAS-FB (test 12); and lactose. (**d**) XRPD patterns of raw PTX, SAS (test 18); SAS (TPGS) (test 19); lactose, SAS-FB (test 12); and SAS-FB (TPGS) (test 13). Conditions for individual tests are shown in Table 1.

The DSC thermograms of all samples are shown in Figure 4a. PTX exhibited an endothermic peak at 224.66 °C, consistent with prior research findings [40]. Lactose, on the other hand, displayed two heat absorption peaks at 145.15 °C and 215.45 °C. Post coating with PTX, both the SAS-FB and SAS-FB (TPGS) samples exhibited heat absorption peaks similar to pure lactose. There was no distinct heat absorption peak specific to PTX attributed to PTX’s low loading capacity and the relatively weak intensity of its heat absorption peak, which is consistent with earlier reports [30]. To further understand the state of PTX on the lactose surface, SAS and SAS (TPGS) samples were prepared without adding lactose. Analysis revealed single endothermic peaks at 225.75 °C and 223.83 °C, respectively, indicating that PTX remained in a crystalline state. An intriguing finding was evident in the SAS (Cou-6) sample alone, demonstrating an endothermic peak at a lower temperature of 192.84 °C compared to PTX and Cou-6 (Figure S2). This lower peak indicated the formation of co-crystallization between PTX and Cou-6 within the SAS-FB process. This formation of eutectic crystals holds the potential for the fluorescent tagging of PTX, enabling efficient tracking within cells. Moreover, it serves as a feasible technical reference for the future fluorescent tagging of PTX.

To investigate potential intermolecular interactions between PTX and TPGS, PTX and lactose, as well as PTX and Cou-6 molecules within the SAS-FB process, FT-IR spectroscopy was employed to characterize the samples across the 4000 to 400 cm^−1^ wavelength range (Figure 4b). As expected, PTX showed absorption bands within the 3300–3500 cm^−1^ range, associated with O–H stretching vibrations. Additional bands observed at 2945 cm^−1^, 1736 cm^−1^, 1705 cm^−1^, and 1646 cm^−1^ confirmed the presence of C–H and C=O stretching vibrations characteristic of PTX, consistent with prior research findings [41]. The FT-IR spectra of the SAS samples was similar to those of PTX, indicating that the SAS process did not impact the chemical or molecular structure of PTX. Similarly, the FT-IR spectrum of the SAS (Cou-6) sample closely resembled that of raw PTX (Figure S3), suggesting that PTX and Cou-6 underwent no chemical reactions during the SAS treatment. Furthermore, it is worth noting that a red shift in the stretching vibration of -CH_2_ at 2888 cm^−1^ in PTX within the SAS (TPGS) sample. This shift might be attributed to potential chemical interactions between hydroxyl groups, influencing the stretching vibration of -CH_2_.

The DRS spectrum results showed the absorption peaks of PTX, lactose, SAS-FB, and SAS-FB (TPGS) samples (Figure 4c). PTX exhibited absorption peaks at 247 nm and 310 nm, while lactose showed peaks at 223 nm and 284 nm. In the SAS-FB samples, absorption peaks were observed at 238 nm, 279 nm, and 309 nm, and in the SAS-FB (TPGS) samples, peaks appeared at 238 nm, 278 nm, and 311 nm. Considering the substantial presence of lactose in the SAS-FB and SAS-FB (TPGS) samples, the absorption peak at 238 nm corresponded to the lactose molecule’s 223 nm absorption peak, showing a considerable red shift. This shift suggested the introduction of auxochrome groups or chromophores from PTX into the lactose molecule, resulting in the formation of new chemical bonds. Moreover, the absorption peaks at 309 nm and 311 nm observed in the SAS-FB and SAS-FB (TPGS) samples were likely linked to the 310 nm absorption peak of PTX. This implies the presence of PTX within these samples. Therefore, the high drug yields achieved in the SAS-FB and SAS-FB (TPGS) samples, reaching 96.8% and 95.0%, respectively, under optimized SAS-FB process conditions, can be attributed to both physical and chemical adsorption interactions between PTX and lactose molecules.

The XRPD patterns shown in Figure 4d demonstrate distinct diffractograms for crystalline PTX, displaying multiple crystallization peaks at various diffraction angles (2 Theta). In contrast, the XRPD patterns of the SAS-FB and SAS-FB (TPGS) samples showed limited variation compared to lactose, attributable to the low drug loading. To assess the crystallinity of samples prepared through the SAS-FB process, SAS particles were generated under identical SAS-FB process parameters but without lactose. Interestingly, we observed a significant reduction or even disappearance of peak intensities at 9.97°, 12.44°, 15.46°, 25.14°, 28.64°, and 29.84° for the SAS and SAS (TPGS) samples compared to unprocessed PTX. This reduction indicated that PTX existed in a microcrystalline state within these samples. Furthermore, the crystallization peak intensity of the SAS (TPGS) sample exhibited a further decrease, suggesting that TPGS had a synergistic effect in inhibiting crystallization during the SAS-FB process. Interestingly, no discernible change in the crystallinity of PTX was observed in the SAS (Cou-6) sample (Figure S4).

The results of the stability test showed that compared to the dissolution of the samples stored for 0 months (Figure 2), the dissolution of the samples stored for 3 months under accelerated stability conditions (Figure S5) all showed a small decrease in dissolution, but there was no difference in the overall change. The dissolution of SAS-FB (TPGS) was the most stable. The results in Figure S6 show that the dissolution of SAS-FB, SAS FB (TPGS), SAS-FB (Gelucire 44/14), and SAS-FB (Pluronic F68) samples remained essentially unchanged in terms of drug content after being stored under accelerated stability conditions for 3 months. The experiments showed that the PTX content as well as the solubility of the samples prepared by the SAS-FB technique had good stability.

### 2.4. Cytotoxicity Studies

The MTT assay results showed intriguing findings regarding the cytotoxicity of PTX, SAS-FB, and SAS-FB (TPGS) samples (Figure S7). Raw PTX and SAS-FB demonstrated low cytotoxicity across various PTX concentrations, with Caco-2 cell survival rates consistently over 90%. However, the SAS-FB (TPGS) sample revealed a reduced Caco-2 cell survival at PTX concentrations exceeding 10 μg/mL. Specifically, the cell survival rate was 80.1% at a PTX concentration of 20 μg/mL and further decreased to 66.3% at 40 μg/mL. No significant toxicity to Caco-2 cells was observed at PTX concentrations below 20 μg/mL. This enhanced cytotoxicity observed in the SAS-FB (TPGS) sample at higher PTX concentrations might be due to TPGS’s capability to inhibit P-gp-mediated transport, subsequently enhancing the internalization of PTX within cells [36,42].

### 2.5. Cellular Uptake Studies

**Figure 5:**
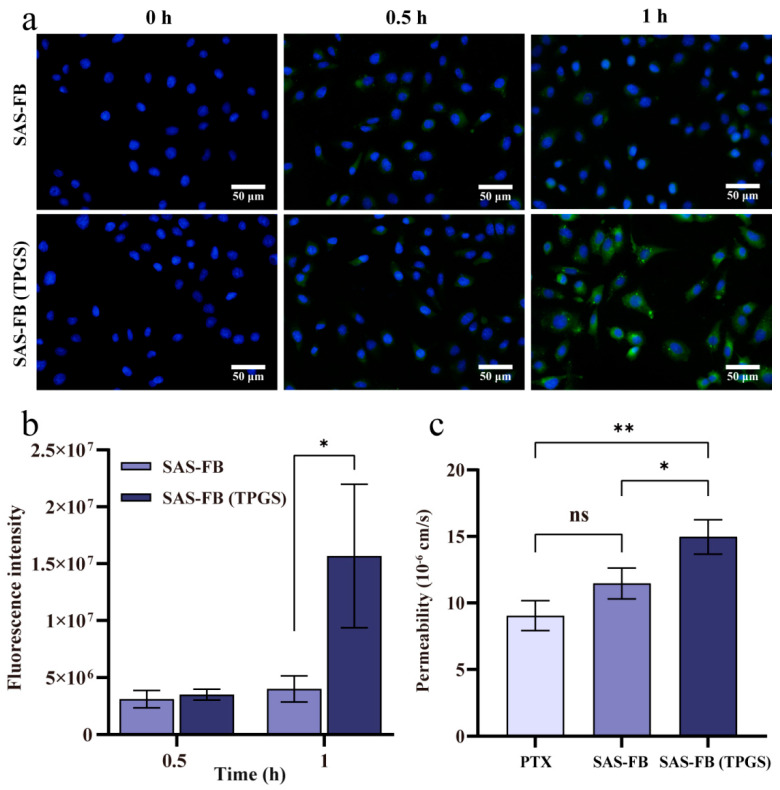
(**a**) Fluorescence microscopic images of Caco-2 cells after 0, 0.5, and 1 h of incubation with coumarin-6 labeled SAS-FB (Cou-6) and SAS-FB (Cou-6,TPGS) at 37 °C, which were imaged by the combined DAPI channel and GFP channel, scale bar = 50 µm; (**b**) analysis of cellular uptake of Caco-2 cells at 0.5 h and 1 h using Image J software (*n* = 3, mean ± SD); (**c**) permeability test. The raw PTX, SAS-FB and SAS-FB (TPGS) solutions were applied in a permeability test in Caco-2 cell monolayers for 4 h at 37 °C (*n* = 3, mean ± SD). ** *p* < 0.01; * *p* < 0.05; ^ns^ *p* > 0.05.

The cellular uptake experiments aimed to assess the differences in PTX internalization between SAS-FB and SAS-FB (TPGS) samples. Since PTX does not have fluorescent properties, fluorescent dye tagging was used for intracellular tracking. In our study, the SAS-FB technique facilitated the labeling of PTX for the cellular uptake assay by co-crystallizing PTX with the fluorescent marker, Cou-6, under supercritical antisolvent conditions (Table 1; tests 16 and 17). Notably, the DSC results (Figure S2) indicated the formation of a eutectic structure between PTX and Cou-6. The results of cellular uptake, visualized through fluorescence microscopy (Figure 5a), showed green fluorescence indicative of PTX internalization in Caco-2 cells. At 1 h of cellular uptake, the green fluorescence of SAS-FB (TPGS) was markedly higher than that of SAS-FB. This enhancement could be due to TPGS’s capability to inhibit P-gp-mediated transport, consequently enhancing drug internalization within cancer cells [32,43]. The further analysis of these results using ImageJ 1.52a software (Figure 5b) showed limited differences in fluorescence intensity between SAS-FB and SAS-FB (TPGS) in Caco-2 cells at the 0.5 h interval. However, at 1 h, a significant difference in fluorescence intensity between SAS-FB and SAS-FB (TPGS) samples within Caco-2 cells was observed (*p* < 0.05), suggesting that the inclusion of TPGS promotes the intracellularization of PTX, enhancing cellular uptake [44].

### 2.6. Transport Assay

Based on the above results, it was initially established that the co-precipitation of TPGS and PTX increased the accumulation of PTX within Caco-2 cells. To further evaluate the transmembrane transport capacity of the SAS-FB and SAS-FB (TPGS) samples, Caco-2 monolayers were selected to mimic intestinal epithelial cells. Higher P_app_ values indicate the increased permeability of the sample across the intestinal barrier, leading to more PTX entering the bloodstream and potentially enhancing oral bioavailability. The PTX dosing concentration chosen for this evaluation was 10 μg/mL. The P_app_ results for the transmembrane transport of PTX, SAS-FB, and SAS-FB (TPGS) samples are shown in Figure 5c. Notably, the P_app_ value of the SAS-FB sample (11.46 ± 0.94 × 10^−6^ cm/s) showed limited differences compared to PTX (9.04 ± 0.91 × 10^−6^ cm/s). This increase in P_app_ compared to previous reports (5.2 × 10^−6^ cm/s) [9] is due to the dissolution of PTX with DMSO and subsequent dilution with HBSS in this study. This similarity in P_app_ values indicates the impact of the P-gp efflux pump. Despite the enhanced solubility and dissolution rate of PTX in the SAS-FB sample due to its smaller particle size, both the SAS-FB sample and PTX remained in the same free state within the transfer medium. Thus, this did not alter the fact that PTX is a substrate for P-gp. However, the P_app_ value of the SAS-FB (TPGS) sample (14.97 ± 1.04 × 10^−6^ cm/s) showed significant differences to both PTX (*p* < 0.01) and the SAS-FB sample (*p* < 0.05), suggesting that the combined effects of the SAS-FB procedure and TPGS altered the transport mode of PTX in the transport media and Caco-2 cells, to some extent inhibiting the efflux of P-gp.

### 2.7. In Vivo Oral Bioavailability

**Figure 6:**
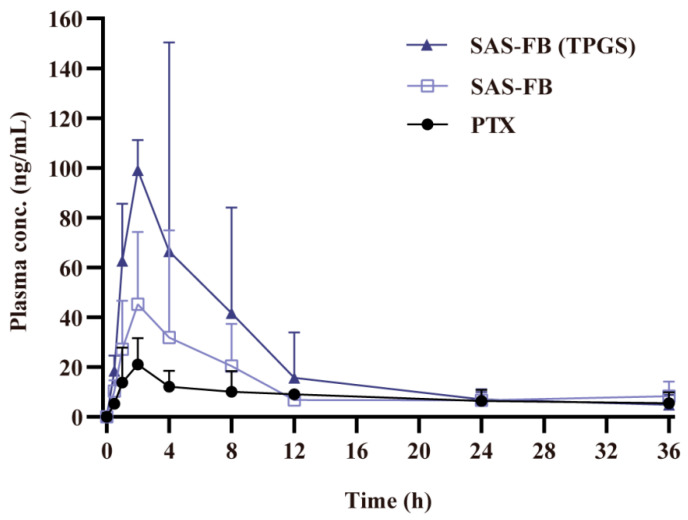
PTX plasma concentration kinetics in rats following oral administration. All formulations were delivered as aqueous suspensions (*n* = 5; mean ± SD).

In order to evaluate changes in PTX levels in plasma following oral administration, SD rats were chosen for the in vivo studies. The pharmacokinetic parameters of the SAS-FB and SAS-FB (TPGS) samples were tested, with PTX as the control. The obtained parameters and mean plasma concentration–time curves post oral administration are presented in Table 2 and Figure 6. 

The SAS-FB (TPGS) sample reached a C_max_ of 99.08 ± 10.80 ng/mL, marking a 4.71- and 2.19-fold increase compared to PTX and the SAS-FB sample, respectively. Meanwhile the AUC_0–∞_ of the SAS-FB (TPGS) sample reached 812.14 ± 336.85 ng/mL, indicating a 2.66- and 1.49-fold increase in bioavailability compared to the PTX and SAS-FB samples, respectively. We further showed that SAS-FB procedure enhances the solubility and dissolution rate of drugs by reducing their particle size from 6.2 μm to 0.7 μm (Figure 3m). In addition, SAS-FB (TPGS) enhances the oral bioavailability of low-solubility and low-permeability PTX. These pharmacokinetic outcomes are consistent and aligned with the results obtained from in vitro dissolution, Caco-2 cellular uptake, and transmembrane transport experiments.

## 3. Materials and Methods

### 3.1. Materials 

Paclitaxel standard (99%) was purchased from Dalian Meilun Biotechnology Co., Ltd. (Dalian, China); paclitaxel (PTX) (98%) was obtained from Xi’an Tongze Biotechnology Co., Ltd. (Xi’an, China); acetonitrile (HPLC grade) and ethanol (A.R. grade) were purchased from Tianjin Tianli Chemical Reagent Co., Ltd. (Tianjin, China); methanol (99.8%) and acetone (99.8%) were obtained from Guangzhou Brand Chemical Reagen Co., Ltd. (Guangzhou, China); Pluronic F68 was obtained from BASF Co., Ltd., (Shanghai, China); Gelucire 44/14 was purchased from MedChemExpress Co., Ltd. (Guangzhou, China); carbon dioxide (99.8 *v*/*v*%) was obtained from Yingxin Gas Co., Ltd. (Guangzhou, China); Lactose Tablettose^®^70, with a particle size range of 150–250 μm, was obtained from Meggle (Shanghai, China); D-α-tocopherol polyethyleneglycol 1000 succinate (TPGS) and 3-(4,5-dimethylthiazol-2-yl)-2,5-diphenyltetrazolium bromide (MTT) were purchased from Sigma Aldrich (Beijing, China); Coumarin-6 (Cou-6) was obtained from Shanghai Aladdin Reagent Co., Ltd. (Shanghai, China); fetal bovine serum (FBS) was obtained from Shanghai ExCell Biotechnology Co., Ltd. (Shanghai, China); 4% paraformaldehyde and 4′,6-diamidino-2-phenylindole (DAPI) were purchased from Shanghai Biyuntian Biotechnology Co., Ltd. (Shanghai, China); Hank’s balanced salt solution (HBSS) was obtained from Beijing Biosharp Technology Co., Ltd. (Beijing, China); penicillin–streptomycin solution, Trypsin-EDTA solution, and DMEM were purchased from Invitrogen Trading (Shanghai) Co., Ltd. (Shanghai, China). Caco-2 cells were obtained from the cell bank of Chinese Academy of Sciences (Beijing, China).

### 3.2. SAS-FB Coating Procedure and Optimization

PTX coating was conducted following the SAS-FB procedure as previously described [26]. In brief, 2 g of lactose carrier particles were placed in a conical glass container to form a fluidized bed. A double-layer filter paper was positioned atop the container to prevent particle dispersion during fluidization. To ensure uniform SC-CO_2_ flow, a stainless steel sieve plate was set at the bed’s bottom. The assembly was subsequently enclosed in a high-pressure vessel (HPV). Compressed CO_2_ was introduced gradually from the bottom simultaneously. Hot water circulating through the autoclave jacket was used to increase the HPV temperature to the set value. Upon achieving stable CO_2_ flow, temperature and pressure, pure solvent was injected into the bed to stabilize experimental conditions at a rate of 1.5 mL/min. Once equilibrium was reached, the liquid feed shifted from solvent to the drug solution at the same rate to initiate the SAS-FB process, coating the antisolvent product onto the carrier particles. Post-processing involved purging pipelines with pure solvent and fluidizing the PTX-coated particles with CO_2_ for an additional 20 min to remove residual organic solvent. After reducing HPV pressure to ambient, the SAS-FB sample granules in the glass container were collected for subsequent analysis. For SAS sample preparation, the same procedure was followed excluding lactose carrier particles. For all experiments, the total mass of the drug used remained constant at 50 mg per run. Table 1 details the experimental parameters and formulations for SAS-FB and SAS granules. Under optimized SAS-FB conditions, samples of PTX processed without any co-coating excipients (test 12) were named SAS-FB and the TPGS and PTX co-precipitated samples were named SAS-FB (TPGS). The ratio of TPGS and PTX in all co-precipitation tests was fixed at 1:4 (*w*/*w*).

### 3.3. Quantitative Determination of PTX Yield

The PTX yield refers to the ratio of mass of the drug coated on the lactose carrier surface to the total mass of drug used in the test. The PTX loading in products of SAS-FB process (SAS-FB sample) was determined using a UHPLC (Ultimate 3000, Thermo Scientific, Guangzhou, China) and the Symmetry C18 column (4.6 × 250 mm, 5 μm, Waters, Shanghai, China) by dissolving the PTX of SAS-FB sample in acetonitrile to obtain the PTX solution, measuring the absorbance of the solution at a wavelength of 227 nm, and comparing with the standard curve (*R*^2^ = 0.9998). The yield of the PTX on the carrier was then estimated by the following formulas [27]:

$$m_{p}=m_{c}\times m_{s}$$

$$drug\;yield(\%)=\frac{m_{p}}{m_{i}}$$

where $m_{c}$, is the actual measured value of sample drug content, in the unit mass sample; m_s_ is the sample mass, total mass of the coated drugs, excipients (TPGS, Pluronic F68, Gelucire 44/14, Cou-6), and lactose carriers in one experiment; m_p_ is the total mass of the drug precipitated in one experiment; and m_i_ is the total mass of the drug introduced in one experiment of the SAS-FB process. 

### 3.4. In Vitro Dissolution

The PTX, SAS, PTX-TPGS (physical mixture, PM), SAS-FB, SAS-FB (TPGS), SAS-FB (Gelucire 44/14), and SAS-FB (Pluronic F68) samples (each equivalent to 2 mg of PTX) were processed using a USP paddle dissolution apparatus (RC8MD dissolution tester, Tianda Tianfa Technology Co., Ltd., Tianjin, China), with a stirring speed of 100 rpm in 900 mL pH 6.8 buffer at 37 ± 0.5 °C. At intervals of 1, 5, 10, and 15 min, one-milliliter samples were withdrawn and immediately replaced with an equal volume of dissolution medium. These withdrawn samples were mixed with acetonitrile, vortexed for 2 min, and then filtered through a 0.22 μm nylon membrane for UHPLC analysis. The reported result represents the mean value obtained from three trials for each time point.

### 3.5. Characterizing the Physicochemical Properties of the Samples

#### 3.5.1. Scanning Electron Microscopy (SEM) 

The morphology of the particles was examined through a Tescan Mira4 scanning electron microscope (Tescan, Shanghai, China). Samples were deposited onto double-sided tape and sputtered with gold for 200 s at 0.5 mbar atmosphere before the analysis. The imaging was achieved at 10 kV and 10 mA. The particle size of raw PTX, SAS processed PTX, and PTX coated onto the SAS-FB products was obtained by measuring more than 200 drug particles from different locations and various magnifications using a specialized image analysis software Image J [28].

#### 3.5.2. Fluorescence Microscopy (FM)

The location and distribution of PTX and Cou-6 compounds on lactose carrier were assessed using a fluorescence microscope (Olympus, BX53, Shenzhen, China). The SAS-FB samples of Cou-6 labeling were scattered on the glass slide and the GFP channel of fluorescence microscopy with a 40× mirror was used to analyze the luminescence of the SAS-FB samples.

#### 3.5.3. Differential Scanning Calorimeter (DSC) 

The thermodynamic properties of samples were examined and analyzed using a DSC-60 system (Shimadzu Co., Ltd., Suzhou, China). A 5 mg sample was heated from 50 to 250 °C in an aluminum dish at a heating rate of 10 °C/min under air atmosphere [28].

#### 3.5.4. Fourier Transform Infrared Spectroscopy (FT-IR) 

The comparison of chemical functional groups between the processed samples and PTX was performed using a Spectrum 100 FT-IR spectrometer (PerkinElmer, Shanghai, China) in the range of 400–4000 cm^−1^. Approximately 2 mg of sample and 100 mg of dry KBr were uniformly blended in a dried agate mortar and pressed into a translucent disc. The spectra were composed of 64 scans with a resolution of 4 cm^−1^ at room temperature [27]. 

#### 3.5.5. Diffuse Reflectance Spectroscopy (DRS)

The composition and structural information of the sample were obtained through a UV-VIS/NIR diffuse reflectance spectrometer (Shimadzu, UV-3600 plus, Suzhou, China). A total of 120 mg of pure sample powder was individually weighed and compressed using a tablet press to a thickness of approximately 4 mm. Before the experiment, baseline calibration was performed on the instrument using a pure barium sulfate sample. The pre-compressed sample was placed on the sample stage for analysis within a wavelength range of 200–800 nm and a resolution ratio of 1.0 nm.

#### 3.5.6. X-ray Powder Diffraction (XRPD) 

To determine the crystalline structure, XRPD patterns of the samples were obtained using an X-ray powder diffraction system (Bruker, D8 Advance, Beijing, China). Powdered samples were examined with a beam angle ranging from 5° to 45° and a step size of 0.05°. The generator tension (voltage) and generator current were maintained at 40 kV and 40 mA, respectively [28].

#### 3.5.7. Stability Test

In this study, drug content and in vitro dissolution were used as indicators to evaluate the stability of the samples. The SAS-FB, SAS-FB (TPGS), SAS-FB (Pluronic F68), and SAS-FB (Gelucire 44/14) samples were sealed with aluminum foil, placed in an airtight desiccator, and placed under accelerated stability conditions (25 ± 2 °C, 60 ± 5% RH) for 3 months before being removed to determine the drug content and in vitro solubility. The samples were taken out after 3 months to determine the drug content and in vitro dissolution.

### 3.6. Cytotoxicity

The cytotoxicity of PTX, SAS-FB, and SAS-FB (TPGS) samples in Caco-2 cells was assessed. Caco-2 cells were seeded in 96-well plates at a density of 6 × 10^3^ cells/well and cultured in DMEM medium with 20% (*v*/*v*) FBS and 1% (*v*/*v*) penicillin–streptomycin solution at 37 °C in a 5% CO_2_ and 90% relative humidity incubator (Heal Force, Shanghai, China). After 24 h, the culture medium was replaced and the PTX, SAS-FB, and SAS-FB (TPGS) samples prepared with DMEM as the dissolution medium were applied. The final PTX concentrations were 0.1, 0.5, 1, 2.5, 5, 10, 20, and 40 μg/mL, each concentration with 6 replicates. Following a 12 h incubation, 20 μL of MTT solution (5 mg/mL in PBS) was added to each well and incubated for 4 h. After discarding the medium, 150 μL of DMSO was added to each well, and absorbance at 490 nm was measured using a SpectraMax i3x Multi-mode Microplate Reader (Molecular Devices, Shanghai, China). Relative cell viability (R%) was calculated as follows:

$$R\%=\frac{{absorbance}_{test}}{{absorbance}_{control}}\times 100\%$$

### 3.7. Cellular Uptake of PTX from SAS-FB and SAS-FB (TPGS)

To evaluate the cellular uptake of SAS-FB and SAS-FB (TPGS) samples labeled with Cou-6, Caco-2 cells were seeded at a density of 5 × 10^4^ cells/well in 24-well plates mounted with cell slides. The culture medium was discarded after 24 h, and samples prepared in DMEM at a final concentration of 5 μg/mL of PTX were added to Caco-2 cells and incubated for 0, 0.5, or 1 h. The drug-containing medium was then discarded following three washes with PBS. A total of 200 μL of 4% paraformaldehyde was added to each well to fix the cells following 15 min incubation at room temperature for 15 min. The cell slides were gently extracted from the 24-well plates and back mounted onto slides containing 8 μL of anti-fluorescence quench mounting agent (containing DAPI) and analyzed with Olympus fluorescence microscope (Olympus, BX53, Shenzhen, China).

### 3.8. In Vitro Transport Studies

Caco-2 cells were seeded at 5 × 10^4^ cells/well onto 12 well Transwell inserts (Polycarbonate Membrane Transwell Inserts, Corning Co., Ltd., Shanghai, China) and 1.5 mL of culture medium was added to the basolateral side (BL). The culture medium was changed every 48 h for the first week and every 24 h thereafter for 21 days. Transepithelial electrical resistance (TEER) of the cell monolayers was evaluated with Millicell-ERS instrument (Millipore, Shanghai, China) in DMEM. When the resistance values exceeded 400 Ω cm^2^, the cell monolayer model was successfully established. Before the experiment, the DMEM was removed and the cell monolayers were gently washed three times with 0.5 mL and 1.5 mL of Hank’s balanced salt solution (HBSS) for the apical side (AP) and the BL, respectively. The cell monolayers were then equilibrated with HBSS for 30 min in the incubator. The HBSS on both sides were removed, and 0.5 mL of test suspensions was diluted in HBSS to achieve a final concentration of PTX of 10 μg/mL, then added to the AP while 1.5 mL of blank HBSS was added to the BL. After 4 h, 200 μL of the sample was aspirated on the BL, and an equal volume of blank HBSS was immediately added to the BL. Three replicate wells were used in each group. The cumulative amount of transported PTX was determined using UHPLC. The apparent permeability coefficients (P_app_) for PTX were calculated according to the following equation:

$$P_{app}=(dQ/dt)/(A\times C)$$

where dQ/dt (mg/s) was the drug permeation rate, A was the surface area of polycarbonate membrane (1.12 cm^2^), and C (μg/mL) was the initial concentration of PTX in the donor compartment (mg/cm^3^).

A total of 200 μL of each sample was vortexed with 800 μL of ethyl acetate for 3 min following centrifugation at 3000× *g* for 10 min, and the upper organic solvent layer was transferred to another new centrifuge tube. The procedure was repeated by adding 800 μL of ethyl acetate to the lower layer. The upper organic solvent layer was confluent into the same centrifuge tube and evaporated to dryness under nitrogen protection. The dried residue was mixed with 100 μL of acetonitrile, vortexed and centrifuged at 12,000× *g* for 5 min, and the supernatant was injected into UHPLC for analysis.

### 3.9. In Vivo Oral Bioavailability and Statistical Analysis

Male Sprague Dawley (SD) rats (body weights of 200–250 g) were purchased from Guangzhou Ruige Biological Technology Co., Ltd. (Guangzhou, China). Pharmacokinetic experiment was approved by Guangzhou University of Chinese Medicine (Guangzhou, China; approval number: DS20230042). After an overnight fast of over 12 h except for free access to water, rats were randomly divided into three groups, with five in each group. Each group received oral doses (10 mg/kg) of PTX, SAS-FB, and SAS-FB (TPGS), respectively. Prior to dosing, each sample was freshly dispersed in a 0.5% sodium carboxymethyl cellulose solution. Blood samples (approximately 0.5 mL) were collected from jugular veins at 0.5, 1, 2, 4, 8, 12, 24, and 36 h post-administration. Following the centrifugation of the blood samples at 4000 rpm for 10 min, the plasma was separated and stored at −80 °C. For analysis, 100 μL plasma, 10 μL acetonitrile, and 10 μL of a 2.5 μg/mL docetaxel solution (internal standard) were combined in a 1.5 mL centrifugal tube and vortexed for 3 min. Subsequently, 1 mL of ethyl acetate was added, vortexed for 5 min, and centrifuged at 12,000 rpm for 10 min. The resulting supernatant (900 μL) was removed, evaporated to dryness under a nitrogen environment, and then redissolved with 100 μL acetonitrile. After vortexing for 2 min and centrifugation at 12,000 rpm for 10 min, the PTX concentration was determined using Accela LC-MS (Thermo Scientific Inc., San Jose, CA, USA). 

Fluorescence intensity, P_app_ value, and pharmacokinetic parameters were compared among the samples using IBM^®^ SPSS^®^ Statistics 26.0 (IBM Corporation, Armonk, NY, USA) analysis software. When the data results had a normal distribution, the data results were expressed as the mean ± SD, and the two sets of measurement data that had a normal distribution and a homogeneity of variance were tested by two independent sample *t*-tests; otherwise, a nonparametric test was used.

## 4. Conclusions 

Our investigation into the parameters of the SAS-FB process, including temperature, pressure, solution concentration, solvent, and excipient, has unveiled an optimal condition for PTX to achieve a coating efficiency of up to 96.8%. Notably, the incorporation of TPGS into the drug solution, facilitating the co-precipitation of PTX and TPGS, resulted in a significant boost in PTX transport by 165.6%, accompanied by a 2.66-fold increase in its bioavailability. Our study demonstrates the capability of SAS-FB technology in enhancing the bioavailability of a BCS class IV drug, thereby expanding the potential applications of this method. By addressing challenges related to dissolution and transmembrane transport, this technology introduces a novel approach to improving oral absorption and bioavailability. Its impact extends beyond the PTX, offering a promising avenue for the development of other BCS class IV drugs. In essence, SAS-FB technology emerges as a promising tool that not only elevates the performance of a specific drug but also paves the way for advancements in the broader landscape of challenging pharmaceutical formulations.

## Figures and Tables

**Table 1:** Experimental parameters. Each experiment used 50 mg of PTX at a 1.5 mL/min drug flow rate and a 32 g/min CO_2_ flow rate.

Exp. No.	T °C	*p* Bar	Solvent	C_drug_ mg/mL	Excipient/Drug (*w*/*w*)	Lactose g	Coating Efficiency %	SD %	Sample Name
1	35	80	Acetone	2	-	2	75.3	1.9	-
2	40	80	Acetone	2	-	2	79.1	1.0	-
3	45	80	Acetone	2	**-**	2	89.1	0.9	SAS-FB (ACE)
4	45	100	Acetone	2	-	2	79.0	6.2	-
5	45	120	Acetone	2	-	2	69.1	5.6	-
6	45	80	Acetone	2.5	-	2	85.6	3.0	-
7	45	80	Acetone	5	-	2	73.8	3.3	-
8	45	80	MeOH	2	-	2	47.2	4.8	SAS-FB (MeOH)
9	45	80	DCM	2	-	2	71.9	9.1	SAS-FB (DCM)
10	45	80	EtOH	2	-	2	82.3	7.0	SAS-FB (EtOH)
11	45	80	ACE-DCM	2	-	2	87.3	4.6	SAS-FB (ACE-DCM)
12 *	45	80	Acetone	1	-	2	96.8	0.2	SAS-FB
13	45	80	Acetone	1	TPGS (1:4)	2	95.0	1.4	SAS-FB (TPGS)
14	45	80	Acetone	1	Gelucire 44/14 (1:4)	2	88.9	1.0	SAS-FB (Gelucire 44/14)
15	45	80	Acetone	1	Pluronic F68 (1:4)	2	88.1	1.4	SAS-FB (Pluronic F68)
16	45	80	Acetone	1	Cou-6 (2:5)	2	85.5	1.3	SAS-FB (Cou-6)
17	45	80	Acetone	1	TPGS/ Cou-6 (1:4/2:5)	2	83.6	1.7	SAS-FB (Cou-6,TPGS)
18	45	80	Acetone	1	-	-	-	-	SAS
19	45	80	Acetone	1	TPGS (1:4)	-	-	-	SAS (TPGS)
20	45	80	Acetone	1	Cou-6 (2:5)	-	-	-	SAS (Cou-6)
21	45	80	Acetone	0	-	2	-	-	Lactose

Note: * The optimal conditions obtained from tests 1 to 11. ACE-DCM: acetone–dichloromethane (50/50, *v*/*v*).

**Table 2:** Pharmacokinetic parameters of PTX, SAS-FB, and SAS-FB (TPGS) after oral administration in rats.

Parameters	PTX	SAS-FB	SAS-FB (TPGS)
t_1/2_ (h)	14.31 ± 2.21	17.02 ±5.00	14.26 ± 3.96
T_max_ (h)	2.00 ± 2.23	2.00 ± 2.69	2.00 ± 2.34
C_max_ (ng/mL)	21.01 ± 9.52	45.22 ± 25.98	99.08 ± 10.80 **
AUC_0→∞_ (ng/mL × h)	305.17 ± 60.16	456.67 ± 226.13	812.14 ± 336.85 **
Relative bioavailability	1	1.49	2.66

Note: Each value represents the mean ± SD (*n* = 5). ** *p* < 0.01 compared to raw PTX.

## Data Availability

Data is contained within the article and Supplementary Material.

## Supplementary Materials

The following supporting information can be downloaded at: https://www.mdpi.com/article/10.3390/ph17040412/s1, Figure S1: Particle size distribution derived from the analysis of scanning electron microscope (SEM) images; Figure S2: Differential Scanning Calorimeter (DSC) heat flow curves of PTX, SAS (Cou-6) (test 20), Cou-6; Figure S3: Fourier Transform Infrared Spectroscopy (FT-IR) spectra of PTX, SAS (Cou-6) (test 20), Cou-6; Figure S4: XRPD patterns of PTX, SAS (Cou-6) (test 20), Cou-6; Figure S5: Dissolution profiles of SAS-FB (Pluronic F68) (test 15), SAS-FB (Gelucire 44/14) (test 14), SAS-FB (test 12), SAS-FB (TPGS) (test 13) in pH 6.8 dissolution media (*n* = 3, mean ± SD); Figure S6: Comparison of drug yield of SAS-FB (Pluronic F68) (test 15), SAS-FB (Gelucire 44/14) (test 14), SAS-FB (TPGS) (test 13), SAS-FB (test 12) samples stored for 0 months and 3 months under stability conditions (*n* = 6, mean ± SD); Figure S7: Cytotoxicity assay of Caco-2 cells after 12 h incubation with raw PTX, SAS-FB and SAS-FB (TPGS) at 37 °C (*n* = 6, mean ± SD).
