# Supplementary material for: Optimizing Paclitaxel Oral Absorption and Bioavailability: TPGS Co-Coating via Supercritical Anti-Solvent Fluidized Bed Technology
Source: Pharmaceuticals (Basel). 2024 Mar 25;17(4):412. doi: 10.3390/ph17040412 (PMC11054146; doi:10.3390/ph17040412)
Supplement: Supplementary file 1 [file pharmaceuticals-17-00412-s001.zip › pharmaceuticals-2929391-supplementary.pdf]

Optimizing paclitaxel oral absorption and bioavailability: TPGS co-coating via supercritical anti-solvent fluidized bed technology

(Supplementary Information)

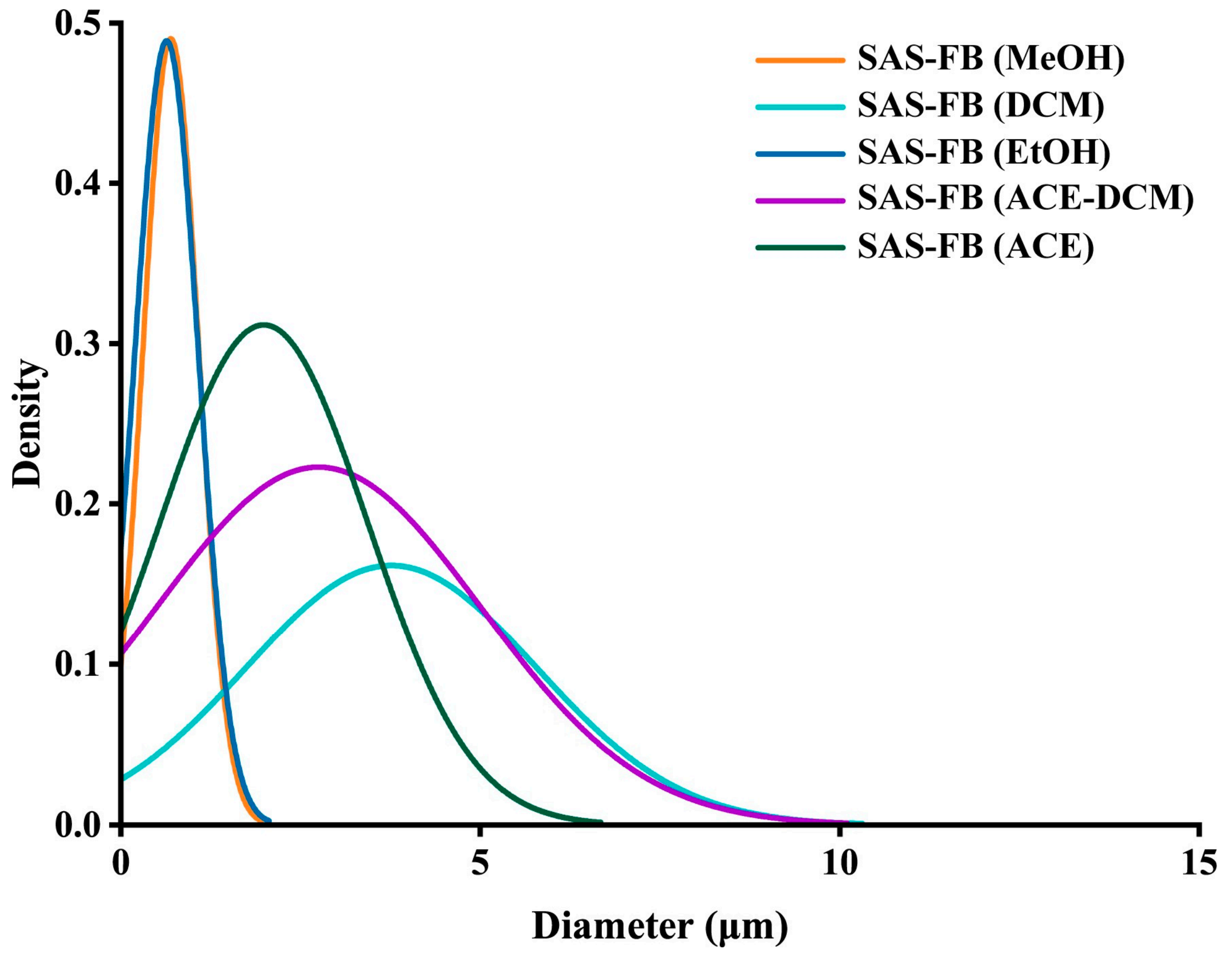

Figure S1. Particle size distribution derived from the analysis of scanning electron microscope (SEM) images.

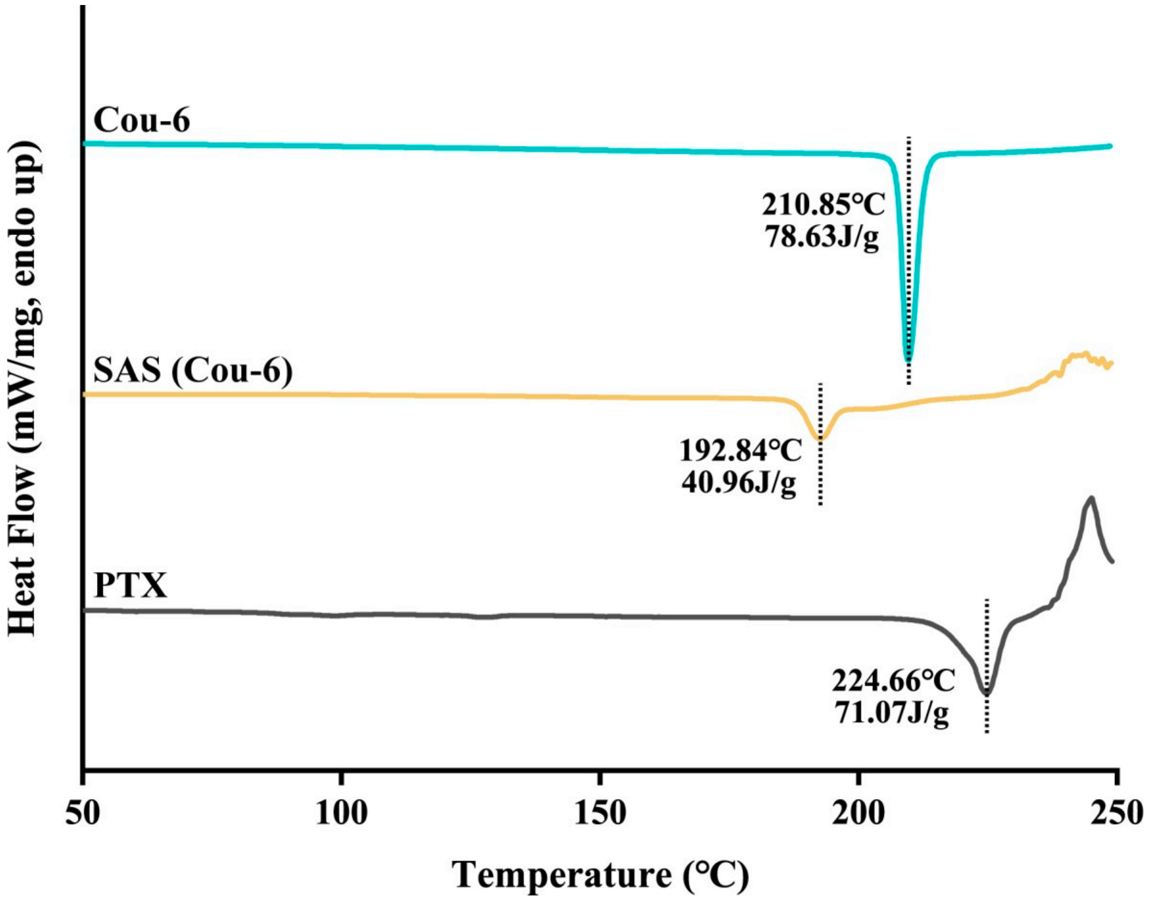

Figure S2. Differential Scanning Calorimeter (DSC) heat flow curves of PTX, SAS (Cou-6) (test 20), Cou-6.

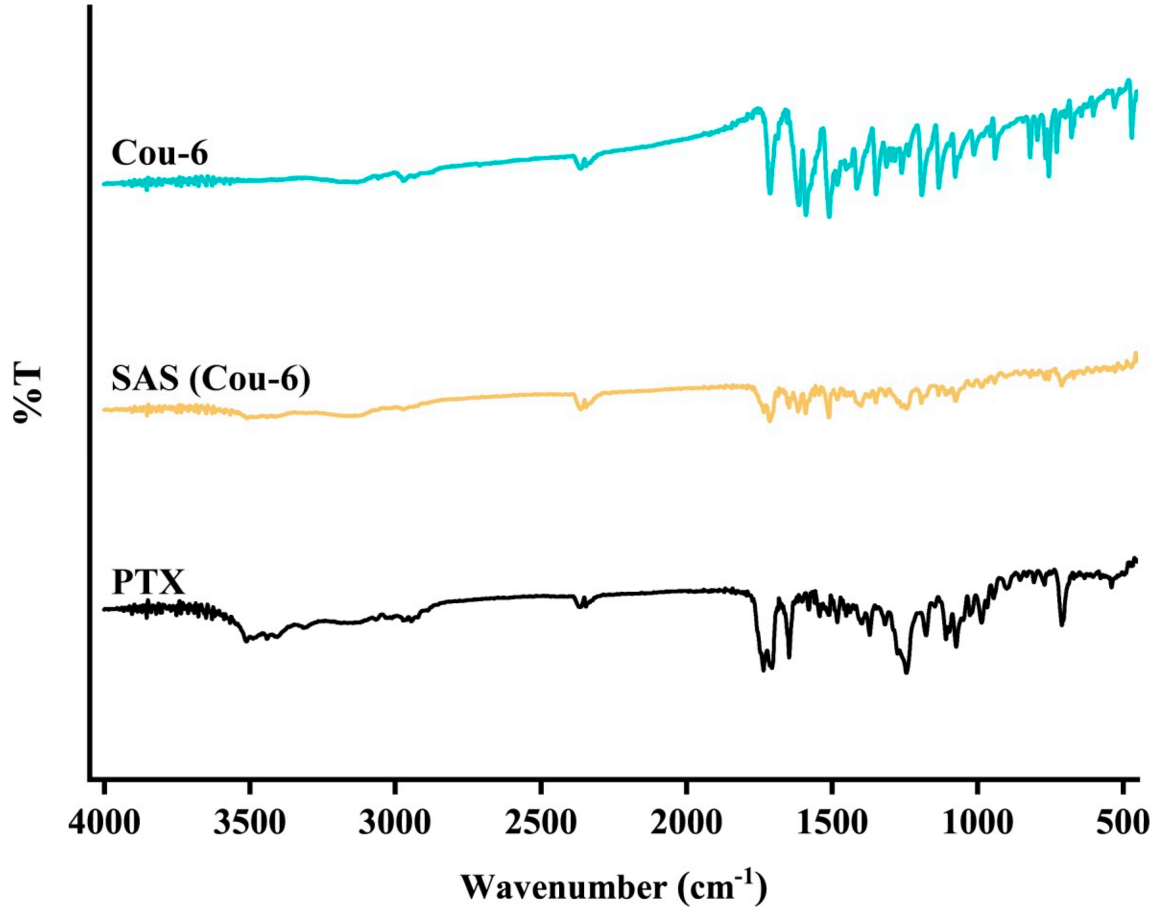

Figure S3. Fourier Transform Infrared Spectroscopy (FT-IR) spectra of PTX, SAS (Cou-6) (test 20), Cou-6.

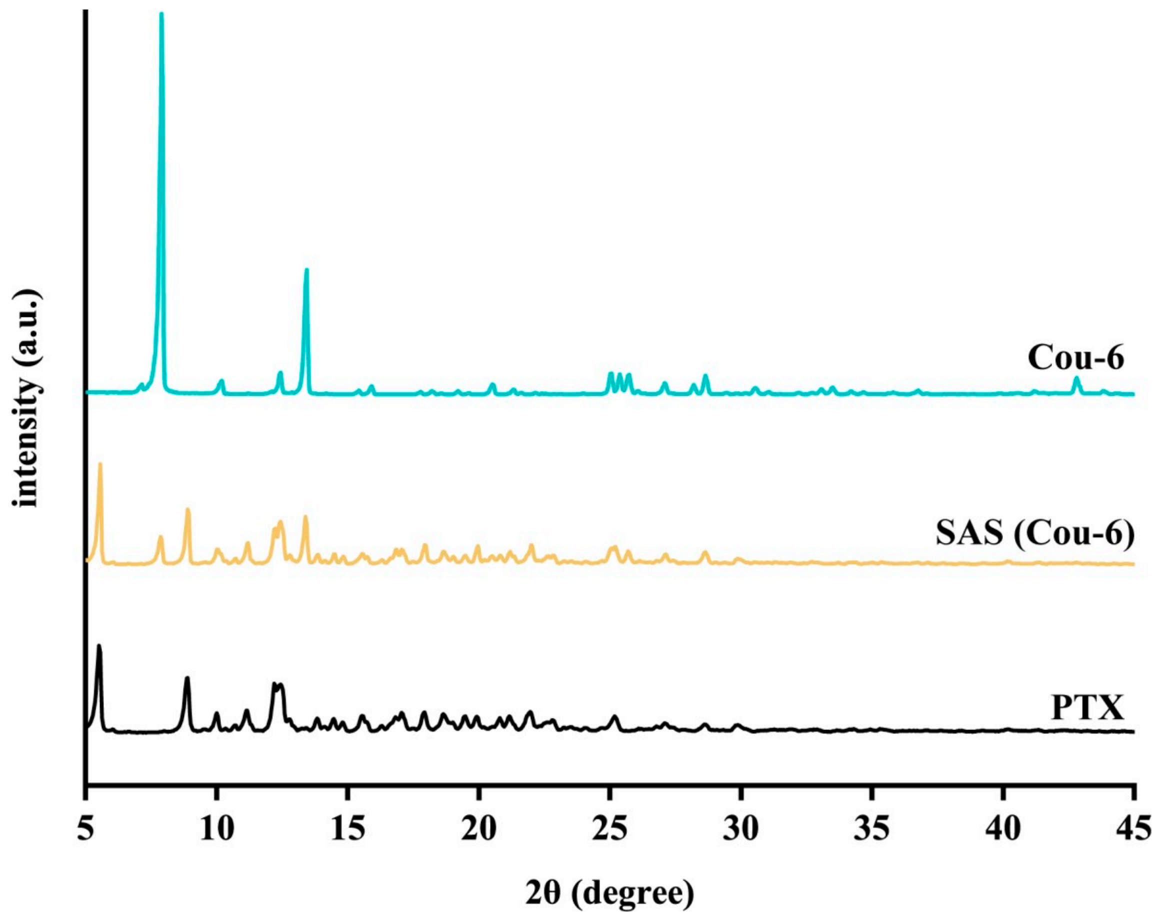

Figure S4. XRPD patterns of PTX, SAS (Cou-6) (test 20), Cou-6.

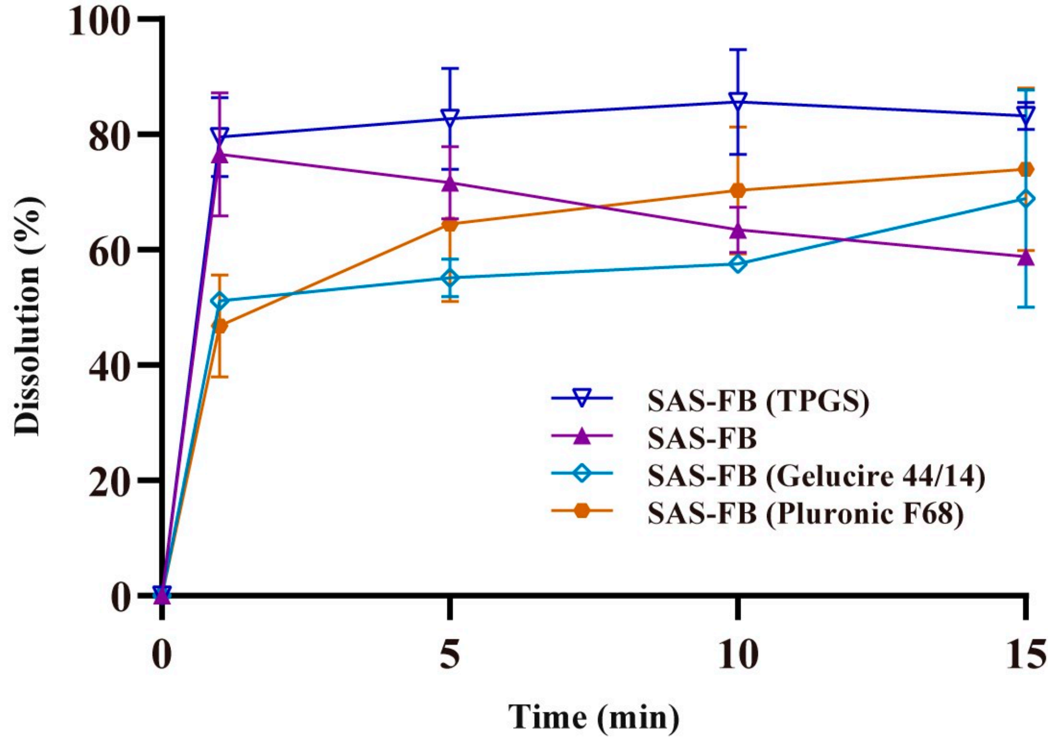

Figure S5. Dissolution profiles of SAS-FB (Pluronic F68) (test 15), SAS-FB (Gelucire 44/14) (test 14), SAS-FB (test 12), SAS-FB (TPGS) (test 13) in pH 6.8 dissolution media (n = 3, mean ± SD).

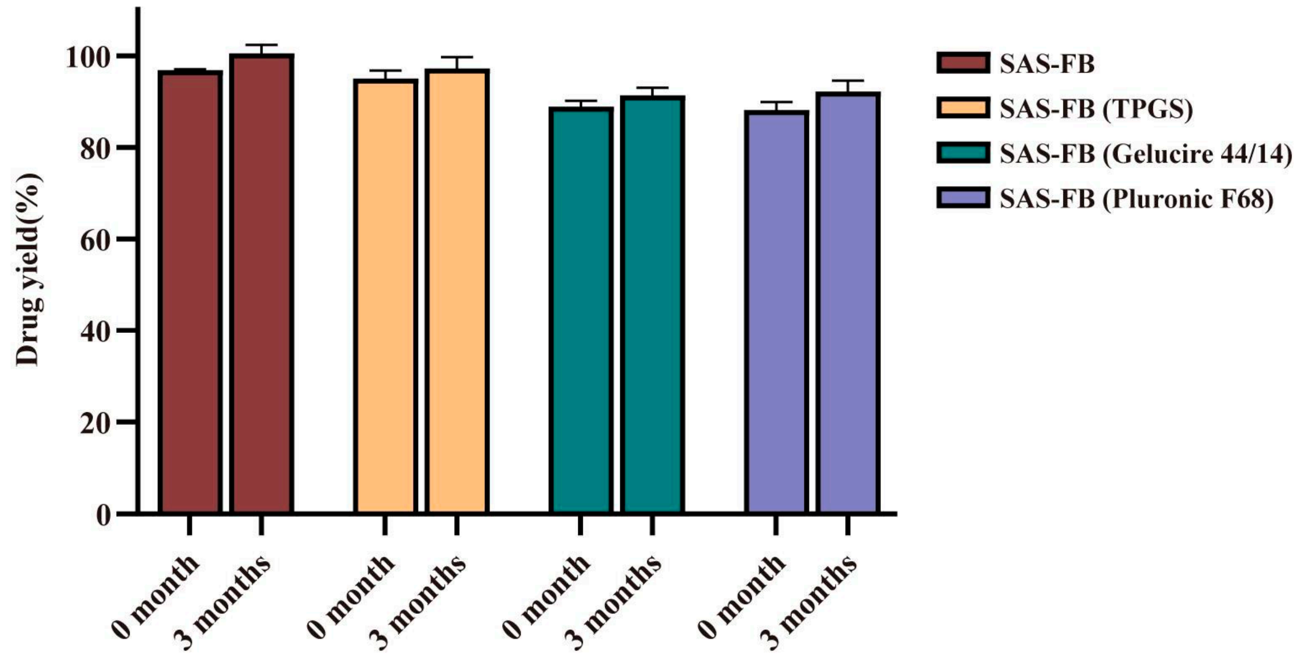

Figure S6. Comparison of drug yield of SAS-FB (Pluronic F68) (test 15), SAS-FB (Gelucire 44/14) (test 14), SAS-FB (TPGS) (test 13), SAS-FB (test 12) samples stored for 0 months and 3 months under stability conditions (n = 6, mean ± SD).

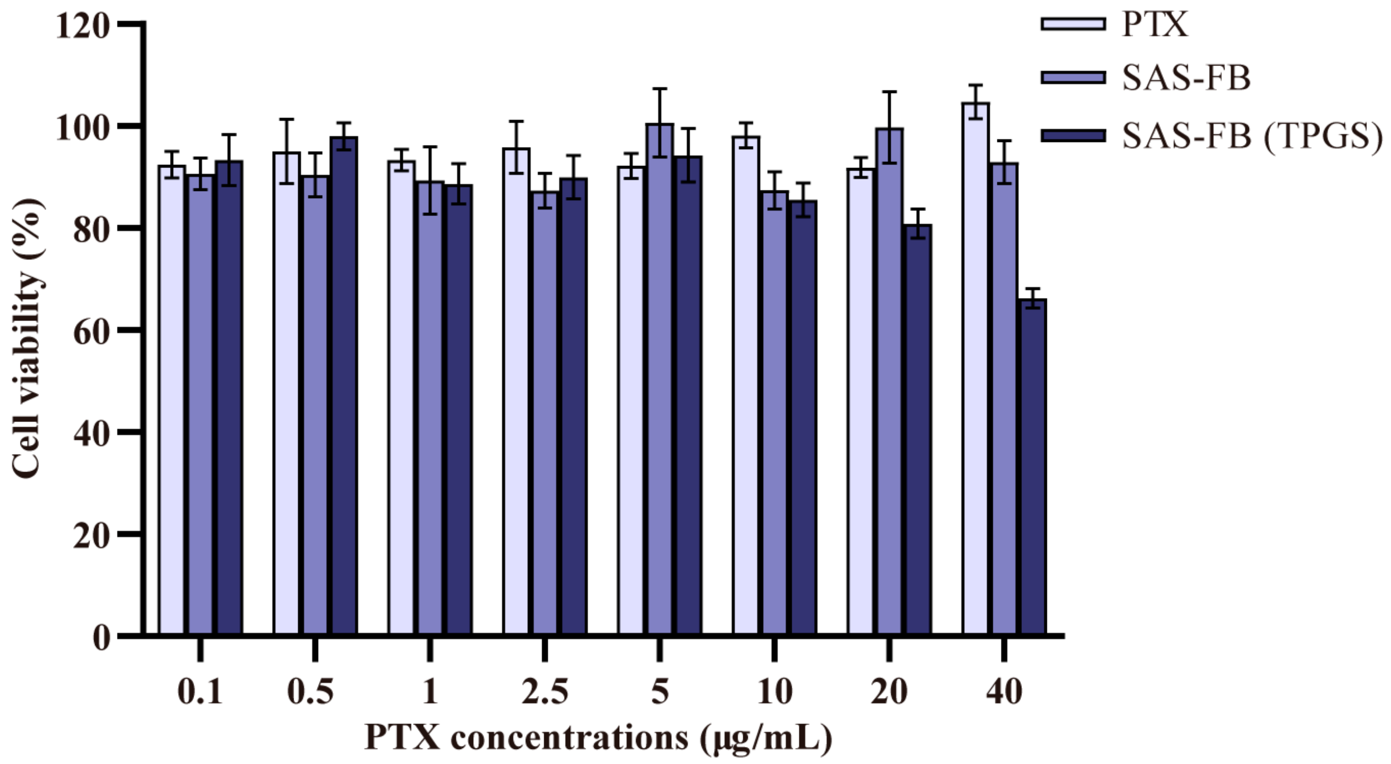

Figure S7. Cytotoxicity assay of Caco-2 cells after 12 h incubation with raw PTX, SAS-FB and SAS-FB (TPGS) at 37°C (n = 6, mean ± SD).
